# Advancing the Study of Positive Psychology: The Use of a Multifaceted Structure of Mindfulness for Development

**DOI:** 10.3389/fpsyg.2020.01602

**Published:** 2020-07-17

**Authors:** Huy P. Phan, Bing H. Ngu, Si Chi Chen, Lijuing Wu, Sheng-Ying Shi, Ruey-Yih Lin, Jen-Hwa Shih, Hui-Wen Wang

**Affiliations:** ^1^School of Education, University of New England, Armidale, NSW, Australia; ^2^Department of Education, National Taipei University of Education, Taipei, Taiwan; ^3^Graduate Institute of Asian Humanities, Huafan University, New Taipei City, Taiwan; ^4^Department of Industrial Engineering and Management Information, Huafan University, New Taipei City, Taiwan; ^5^Department of Buddhist Studies, Huafan University, New Taipei City, Taiwan; ^6^Department of Asian Philosophy and Eastern Studies, Huafan University, New Taipei City, Taiwan

**Keywords:** Buddhism, Confucianism, positive psychology, mindfulness, meditation, optimal best, flourishing, optimization

## Abstract

Positive psychology, as a distinctive paradigm, focuses on the remedy of pathologies and, by contrast, the promotion of positive experiences and conditions in life (e.g., encouraging a state of flourishing). Positive psychology, in its simplistic form, may provide evidence and insightful understanding into the proactivity of human agency (Seligman, 1999; Seligman and Csíkszentmihályi, 2000). Drawing from this emphasis, we have developed the *theory of optimization*, which attempts to explain the achievement of optimal functioning in life (e.g., optimal cognitive functioning: academic performance). By the same token, in the course of our research development into the theory of optimization, we have also delved into a comparable theoretical orientation, namely: the *multifaceted nature of mindfulness*, consisting of three interrelated components – the psychological component of mindfulness, the philosophical component of mindfulness, and the spiritual component of mindfulness. This conceptualization of mindfulness is rather unique for its incorporation of both Western and Eastern knowledge, philosophical viewpoints, and epistemologies into one holistic framework. The main premise of this conceptual analysis article is to advance the study of positive psychology by specifically introducing our recently developed model of mindfulness, in this case, the multifaceted structure of mindfulness with its three distinct components. Importantly, we make attempts to highlight the significance of this multifaceted model by situating it within the theory of optimization for academic learning. Using philosophical psychology and personal-based teaching and research reasoning, we provide a valid rationale as to how aspects of our proposed model of mindfulness (e.g., reaching a state of enlightenment) could act to facilitate and optimize a person’s state of functioning (e.g., cognitive functioning). Moreover, we posit that our rationale regarding mindfulness as a potential “optimizing agent” for the purpose of optimal functioning could, indeed, emphasize and reflect the salient nature of positive psychology. In other words, we contend that an explanatory account of mindfulness from the perspectives of Confucianism and Buddhism could, in this analysis, coincide with and support the meaningful understanding and appreciation for the study of positive psychology in educational and non-educational contexts. We conclude the article by exploring the complex issue of methodology – that is, for example, how would a researcher measure, assess, and/or empirically validate the multifaceted nature of mindfulness?

## Introduction

The present article makes attempts to accentuate the important nature of positive psychology (Csíkszentmihályi, 1990; Seligman, 1999; Seligman and Csíkszentmihályi, 2000) by taking into account and incorporating the theoretical concept of mindfulness. In other words, our main premise is to introduce preliminary details of our recently developed theoretical model of mindfulness (Phan and Ngu, 2019; Phan et al., 2019d), which could hopefully instill appreciation and facilitate meaningful understanding into the paradigm of positive psychology. This theoretical-conceptual article emphasizes the use of philosophical psychology and personal reasoning to rationalize the *potential intricate association between mindfulness and positive psychology*. We contend that our proposed model of mindfulness is innovative and, indeed, espouses notable tenets of Buddhism (e.g., enlightenment) that may, in effect, advance understanding into the true nature of positive psychology.

In the next section of this article, we provide an overview of positive psychology (Csíkszentmihályi, 1990; Seligman, 1999; Seligman and Csíkszentmihályi, 2000), which is then followed by a brief theoretical account of the theory of optimization (Fraillon, 2004; Phan et al., 2017, 2019c) and its association with the concept of optimal best practice (Fraillon, 2004; Martin, 2006, 2011; Phan et al., 2016). This overview in the initial stage is beneficial, forming grounding for the subsequent sections of the article – namely, an examination of the theoretical concept of mindfulness. In the latter section of the article, we offer a conceptualization, which researchers may consider for their own inquiries. One notable line of inquiry, in this case, is related to the development of an appropriate methodological design that could measure, assess, and validate our proposed model of mindfulness.

## The Importance of Positive Psychology: a Brief Overview

There has been extensive research development pertaining to the nature of positive psychology. Why study positive psychology? Positive psychology, emerging within the field of psychology as a paradigm for quality teaching and scientific research development (Csíkszentmihályi, 1990; Seligman, 1999; Seligman and Csíkszentmihályi, 2000), lies in its nature to address and prevent pathologies and maladaptive experiences. Moreover, positive psychology, spanning the course of three decades, is concerned with the encouragement and promotion of positive experiences and conditions in life (Pawelski, 2016). Indeed, according to Gable and Haidt (2005), the study of positive psychology considers different internal and external conditions that could contribute to a person’s and/or an organization’s state of optimal functioning.

So, what is positive psychology? According to Sheldon et al. (2000), positive psychology:

“Positive Psychology is the scientific study of optimal human functioning. It aims to discover and promote the factors that allow individuals and communities to thrive. The positive psychology movement represents a new commitment on the part of research psychologists to focus attention upon the resources of psychological health, thereby going beyond prior emphases upon disease and disorder” (section “The Importance of Positive Psychology: A Brief Overview”).

This definition, as reflected in Pawelski’s (2016) comprehensive review of this topic, connotes the inclusion of attributes such as personal growth, mastery, drive, character building, human strength, and family and civic virtue. From this emphasis, the study of positive psychology may entail the “building of the most positive qualities of an individual” (Seligman, 1999) and “on building of what makes life most worth living” (Seligman, 1999). Seligman and Csíkszentmihályi’s (2000) published work, likewise, emphasizes the science of positive psychology may exist on three levels – subjective, individually, and institutional: “the field of positive psychology at the subjective level is about valued subjective experiences: well-being, contentment, and satisfaction (in the past); hope and optimism (for the future); and flow and happiness (in the present). At the individual level, it is about positive individual traits: the capacity for love and vocation, courage, inter-personal skill, aesthetic sensibility, perseverance, forgiveness, originality, future mindedness, spirituality, high talent, and wisdom. At the group level, it is about the civic virtues and the institutions that move individuals toward better citizenship: responsibility, nurturance, altruism, civility, moderation, tolerance, and work ethic” (p. 5).

From the above, positive psychology is well-balanced in scope delving into the resolution of the spectrum of both negative and positive life experiences (Pawelski, 2016) – that is, flourishing at one end of the continuum (i.e., positive) and languishing at the other (i.e., negative) (Keyes, 2005). In terms of the proactivity of human agency (Phan et al., 2020a), incorporation of positive psychology may involve the fostering of optimal functioning. In this analysis, deep meaningful understanding of positive psychology acknowledges the entirety of human experiences with the hope that we could facilitate, motivate, and enhance optimal conditions for the purpose of self-fulfillment and flourishing. This tenet reflects importantly, from our viewpoint, the maximization of a state of condition or functioning – that is, the notion of optimal condition or functioning in a subject matter or contextual setting.

Recent research development using positive psychology as a main premise has explored an interesting topic, known as optimal functioning (Martin, 2011; Liem et al., 2012; Phan et al., 2016). Optimal functioning, or optimal best practice, is concerned with the *maximization* of a person’s acquired knowledge, experience, and/or personal state of flourishing in a subject matter (e.g., feeling good about oneself) (Ngu et al., 2019). Over the past 5 years, we have made extensive theoretical, methodological, and empirical contributions to the study of optimal best practice, especially within the realm of academia (e.g., Ngu et al., 2019; Phan et al., 2019b, 2020b).

### The Importance of Optimal Best Practice

Optimal best practice is an important topical theme for discussion as it reflects the nature of positive psychology. In brief, from the preceding section, we know that optimal best practice is concerned with the maximization of fulfillment and/or accomplishment – say, in mathematics learning in the topic of Algebra. At the same time, however, achievement of optimal best is indicative of positive psychology, especially in terms of personal experience of fulfillment and inner satisfaction. What is of interest from this understanding, as initially raised by Fraillon (2004), is the methodological account or explanation of how optimal best is calculated or derived. This examination, which we have explored in-depth, is insightful in helping to elucidate the positive nature of positive psychology.

**FIGURE 1:**
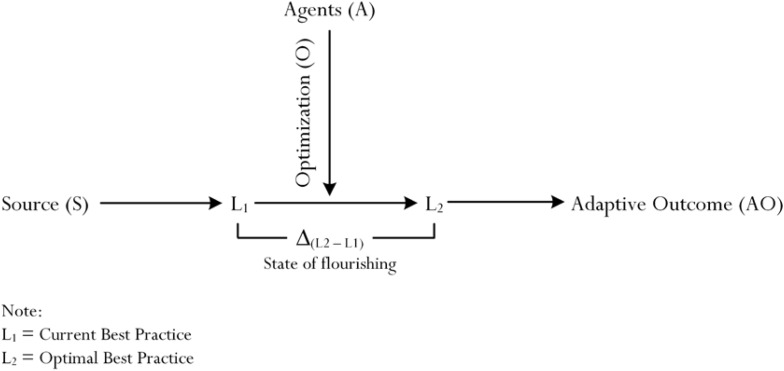
Simplistic representation of process of optimization.

The concept of optimal best practice is significant, especially in light of our focus on the use of mindfulness to appreciate the nature of positive psychology. Fraillon (2004) and Phan and his colleagues (e.g., Phan et al., 2016, 2017) have been prominent in their respective discussions regarding the operational nature of optimal best practice – that is, for example, how does one achieve a state of optimal best practice in a subject matter? According to the authors’ explanations, achievement of optimal best practice requires a point of reference or, alternatively, optimal best practice is intricately linked to a reference point. Phan et al.’s (2019b) recent article is insightful for its detailed account, which we refer in this section. Figure 1 is a summary of the *process of optimization* (Phan et al., 2017, 2019c), which shows two levels of best practice:

i. A level of *current best practice*, denoted as L_1_, according to Fraillon (2004) and Phan et al. (2019c), is defined as a person’s perceived level of functioning at the present time – for example, “what is it that I am capable of at present in Algebra?” (e.g., I am able to solve equations with one unknown, *x*, at present).
ii. A level of *optimal best practice*, denoted as L_2_, in contrast, is defined as a person’s perceived maximum level of functioning that could be fulfilled and/or accomplished (Fraillon, 2004; Phan et al., 2019c) – for example, “I perceive and believe that I am capable of accomplishing….. in Algebra” (e.g., I am capable of solving equations with two unknowns, *x* and *y*. This accomplishment is my maximum capability).

The relationship between L_1_ and L_2,_ in its simplistic term, according to Phan et al.’s (2019b) recent study, is shown in Figure 1. The uniqueness of Figure 1 lies in the concise representation of the enactment of optimization (Phan et al., 2017, 2019c), and a “state of flourishing” – in this case, defined as “a quantitative and qualitative difference between L_1_ and L_2_ [i.e., Δ_(L1–L2)_].” The theory of optimization (Phan et al., 2019c, 2020b) indicates that the achievement of L_2_ from L_1_ would require the activation and enactment of different types of *educational* (e.g., an appropriate instructional design: Ngu et al., 2018), *psychological* (e.g., belief of personal efficacy: Bandura, 1997), and *psychosocial* (e.g., the impact of the home environment: McCartney et al., 2007) agencies. Phan et al.’s (2019c) detailed proposition, interestingly, stipulated the positive effects of educational, psychological, and/or psychosocial agencies on the initiation of experience of “energy” (denoted as “E”), which would then activate the buoyancy of different psychological attributes (e.g., personal resolve). Buoyant psychological experiences, in turn, would arouse and sustain the accomplishment of L_2_.

Optimal best practice, L_2_, may entail different types of functioning – for example, cognitive functioning in a school context may reflect exceptional exceptional academic performance in essay composition. Optimal best practice in emotional development, likewise, may consist of a person’s state of happiness. Aside from the importance of optimal best practice, poignant from Figure 1 also is the description that pertains to a person’s state of flourishing – denoted as Δ_(L2–L1)_. Flourishing in this case, similar to other comparable definitions (e.g., Diener et al., 2010; Huppert and So, 2013), is positive and reflects improvement, accomplishment, and self-fulfillment. From Phan et al.’s (2019c) theory of optimization, there are three main premises for acknowledgment:

i. A focus on the facilitation of L_2_, via means of different types of optimizing agents (e.g., educational agent such as an appropriate instructional design: Ngu et al., 2016).
ii. A focus on ensuring that a person experiences a state of flourishing, which would equate to a positive difference between L_1_ and L_2_.
iii. The underlying role of energy, which is central to the process of optimization, facilitating a person’s improvement and progress from L_1_ to L_2_.

From the preceding sections, one notable aspect of positive psychology (Csíkszentmihályi, 1990; Seligman, 1999; Seligman and Csíkszentmihályi, 2000) is concerned with the accomplishment and fulfillment of optimal conditions and life experiences (e.g., feel-good experience). Since the emergence of positive psychology, there have been different theoretical models developed to help foster the accomplishment and fulfillment of optimal best practice – emotionally, socially, cognitively, physically, and socially. By the same token, of course, constructive models reflecting the importance of positive psychology have also focused on preventive measures, which could resolve and weaken negative life experiences, pathologies, etc. From the literature, for example, there are some interesting models: Seligman’s (2010) *PERMA* model (i.e., Positive Emotions, Engagement, Relationship, Meaning, and Accomplishment), Csíkszentmihályi’s (1990) *flow theory*, Keye’s (2002) *continuum of psychological wellbeing*, Peterson and Seligman’s (2004) *Character Strengths and Virtues Framework*, and Phan et al.’s (2017) *Framework of Achievement Bests*.

Indeed, from a practical point of view, not to mention theoretically, there is impetus for us to consider research development, policies, programs, pedagogical practices, etc. that may assist in the fostering of flourishing. Within the context of schooling, as an example, it is pertinent that educators consider different opportunities, pathways, means, etc. that may assist students in their learning (e.g., mastery experience in a subject matter) and non-learning (e.g., psychological well-being) experiences. In a recent study, for example, Phan et al. (2019a) found that social relationships with others (e.g., peers) and enriched academic experiences may serve to enhance positive emotions. In another similar study, Hasnain et al. (2014) reported that both hope and happiness positively influenced students’ psychological well-being. Tugade and Fredrickson (2007), interestingly, offered a number of strategies that could be considered for usage (e.g., relaxation therapies and meditation practices).

## Introducing the Importance of Mindfulness

Phan et al.’s (2020b) recent article is significant as it introduces the concept of *time* (Frank, 1939; Lewin, 1942; Wallace, 1956; Nuttin, 1964; Mehta et al., 1972) and its potential association with the achievement and fulfillment of L_2_. In this analysis, Phan et al. (2020b) contend that in order for one to achieve a state of flourishing, Δ_(L2–L1)_, he/she would need to structure and have an appropriate future time point (e.g., 6 months). In other words, from the authors’ rationale, achievement of L_2_ from L_1_ does not occur instantaneously but requires adequate time. By the same token, this rationalization also considers the plausibility that a future time orientation may, in itself, serve as a source of motivation, which would direct and compel a person to strive for optimal best. This rationalization is interesting, highlighting the complexity of the fulfillment of L_2_.

Considering Phan et al.’s (2020b) rationalization of time, we consider another related concept, which could serve to facilitate the achievement of optimal best practice: *mindfulness*. What is so unique about mindfulness and why would we would want to include this concept for in-depth examination, especially with reference to the study of positive psychology? There are three major reasons:

i. Our individual and collective interests in mindfulness from an Eastern perspective, which in this case encompasses both Confucianist thinking and Buddhist philosophy. We contend that this article is appropriate, allowing us to introduce our proposition of a theoretical model of mindfulness (Phan and Ngu, 2019; Phan et al., 2019d) for readers to appreciate.
ii. Mindfulness is personal and proactive, coinciding with and reflecting the true nature of positive psychology (Csíkszentmihályi, 1990; Seligman, 1999; Seligman and Csíkszentmihályi, 2000). For example, meaningful understanding of mindfulness may assist a person to feel “enlightened,” resulting in the fulfillment of happiness.
iii. We contend that it would be of interest to consider mindfulness, via means of meditation practice as an optimizing agent, as Phan et al. (2019d) recently discussed in their theoretical-conceptual chapter. In this analysis, as a proposition, we posit that our derived model of mindfulness could optimize a person’s state of functioning.

In this section of the article, we want to explore the definition(s) and scope of mindfulness from a psychological point of view. The latter sections of the manuscript introduce our recently developed model of mindfulness (Phan and Ngu, 2019; Phan et al., 2019d), and how this theorization could indeed explain the specific reference to optimal best practice – in this case, the use of optimization, as an underlying process, to explain for the achievement of L_2_.

### Definition and Scope of Mindfulness and Meditation

Scientific research into the concept of *mindfulness* is well documented in different journals (e.g., the journal of *Mindfulness*). One important line of research development, in this case, focuses on clarity into the *definition* and *nature* of mindfulness. Aside from definition, the “nature” of mindfulness connotes understanding of its scope and underlying structure – that is, what constitutes the “essence” of mindfulness? This question, we contend, reflects a similar theoretical approach to the study of other psychological concepts, such as *self-efficacy* (Bandura, 1977, 1997), *self-concept* (Shavelson et al., 1976; Marsh et al., 1988), and *engagement* (Schaufeli et al., 2002; Fredricks et al., 2004).

There are a number of comparable definitions of mindfulness. For example, Kabat-Zinn (2015) defines mindfulness as “moment-to-moment, non-judgmental awareness, cultivated by paying attention in a specific way, that is, in the present moment, and as non-reactively, as non-judgmentally, and as openheartedly as possible.” Desbordes et al. (2015), differently, makes reference to mindfulness as “the quality of mind that one recollects continuously without forgetfulness or distraction while maintaining attention on a particular [mental] object.” Brown and Ryan (2003), acknowledging Buddhist and contemplative traditions and other researchers’ theoretical contributions, define mindfulness as “a state of being attentive to and aware of what is taking place in the present.” This definition, as the authors noted, reflects both Nyanaponika Thera (1972) (i.e., “the clear and single-minded awareness of what actually happens to us and in us at the successive moments of perception,” p. 5) and Hanh’s (1976) definition of mindfulness (i.e., “keeping one’s consciousness alive to the present reality,” p. 11). From our own teaching, coinciding with Buddhist philosophies and the importance of Confucianism, we surmise mindfulness as being of the following: a person’s state of: (i) *awareness of the present moment*, (ii) *consciousness and focus of his/her contextual surrounding*, and (iii) *concentration of a designated object in mind (e.g., image of Buddha)*.

What actually defines mindfulness, as detailed from the above, in turn reflects the importance of the concept’s nature – that is, its scope and underlying structure. From an empirical point of view, researchers have used a quantitative methodological approach (Hanson et al., 2005; Babbie, 2014), involving both experimental and non-experimental data to study the true nature of mindfulness. For example, non-experimentally, researchers have used factorial techniques to test and compare competing *a priori* and *a posteriori* model (Schumacker and Lomax, 2004; Kline, 2011) – does a one-factor model, in this instance, represent the underlying structure of mindfulness? Or, comparatively, does a two-factor model provide a stronger representation of mindfulness? From the literature, we see that numerous questionnaires (e.g., The Toronto Mindfulness Scale; Lau et al., 2006) have been developed to measure and assess the construct of mindfulness. Table 1 illustrates a brief summation of existing research that has used both open-ended and close-ended questionnaires to gauge into the nature and predictive effect of mindfulness. It is interesting to note that there is no definitive consensus as to what actually constitutes mindfulness. Some researchers, for example, have established a simple structure: a *one-factor* (e.g., Brown and Ryan, 2003; Chadwick et al., 2008) and a *two-factor* (e.g., Cardaciotto et al., 2008; Davis et al., 2009) model. In contrast, too, other researchers tested and established more complex models: a *four-factor* (e.g., Baer et al., 2004; Feldman et al., 2007), a *five-factor* (Baer et al., 2006), and a *six-factor* (Neff, 2003) model.

**TABLE 1:** Mindfulness scales and inventories.

**Scales**	**Age**	**Rating**	**Components**	**sample items**
The Five Facet Mindfulness Questionnaire (e.g., Baer et al., 2006)	Undergraduate Psychology students (*N* = 613)	1 = Never or very rarely true 5 = Very often or always true	*Observing*	1. I sense my body, whether eating, cooking, cleaning, or talking. 2. I notice how my emotions express themselves through my body.
			*Describing*	1. I am good at finding words to describe my feelings. 2. I can easily put my beliefs, opinions, and expectations into words.
			*Acting with awareness*	1. I break or spill things because of carelessness, not paying attention, or thinking of something else. 2. I find myself doing things without paying attention.
			*Non-judging of inner experience*	1. I criticize myself for having irrational or inappropriate emotions. 2. I think some of my emotions are bad or inappropriate and I should not feel them.
			*Non-reactivity to inner experience*	1. I perceive my feelings and emotions without having to react them. 2. I watch my feelings without getting lost in them.
The Mindful Attention and Awareness Scale (e.g., Brown and Ryan, 2003)	Undergraduate students (*N* = 313)	1 = Almost always 6 = Almost never	*One latent factor*	1. I could be experiencing some emotion and not be conscious of it until sometime later.
	General Community adults (*N* = 79)			2. I break or spill things because of carelessness, not paying attention, or thinking of something else.
	Undergraduate Psychology students (*N* = 90)			
The Toronto Mindfulness Scale (e.g., Davis et al., 2009)	General Community (*N* = 369) and First-year Psychology students (*N* = 92)	0 = Not at all 4 = Very much	*Curiosity*	1. I am curious about what I might learn about myself by taking notice of how I react to certain thoughts, feelings or sensations. 2. I am curious to see what my mind is up to from moment to moment.
			*Decentering*	1. I experience myself as separate from my changing thoughts and feelings. 2. I am more concerned with being open to my experiences than controlling or changing them.
The Revised 12-item Cognitive and Affective Mindfulness Scale (e.g., Feldman et al., 2007)	University students (*N* = 548) College students (*N* = 212)	1 = Rarely/Not at all 2 = Sometimes 3 = Often 4 = Almost always	*Present Focus*	1. I am preoccupied by the future. 2. I am able to focus on the present moment.
			*Attention*	1. It is easy for me to concentrate on what I am doing. 2. I am able to pay close attention to one thing for a long period of time.
			*Awareness*	1. I can usually describe how I feel at the moment in considerable detail. 2. I try to notice my thoughts without judging them.
			*Acceptance*	1. I can tolerate emotional pain. 2. I am able to accept the thoughts and feelings I have.
The Southampton Mindfulness Questionnaire (e.g., Chadwick et al., 2008)	Non-Clinical Community (*N* = 134) and Clinical (*N* = 122)	0 = Strongly disagree 6 = Strongly agree	*One latent factor*	1. I am able just to notice them without reacting. 2. I judge the thought/image as good or bad.
The Philadelphia Mindfulness Scale (e.g., Cardaciotto et al., 2008)	Undergraduate Psychology students (*N* = 204) Undergraduate Psychology students (*N* = 559) Clinical patients (*N* = 52) Clinical patients (N = 30) Graduate students (*N* = 78) in Health Programs	0 = Never 1 = Rarely 2 = Sometimes 3 = Often 4 = Very often	*Awareness*	1. I am aware of what thoughts are passing through my mind. 2. When talking with other people, I am aware of their facial and body expressions.
			*Acceptance*	1. I try to distract myself when I feel unpleasant emotions. 2. There are aspects of myself I don’t want to think about.
The 30-item Freiburg Mindfulness Inventory (e.g., Sauer et al., 2011)	British patients (*N* = 130)	1 = Almost never 4 = Almost always	*Presence*	1. I pay attention to what’s behind my actions. 2. I am open to the experience of the present moment.
			*Acceptance*	1. I watch my feelings without getting lost in them. 2. I am able to appreciate myself.
The Kentucky Inventory of Mindfulness Skills Scale (e.g., Baer et al., 2004)	Undergraduate Psychology students (*N* = 205), Undergraduate Psychology students (*N* = 215), and Adults with borderline personality disorder (*N* = 26)	1 = Never or very rarely true 5 = Almost always or always true	*Acting with awareness*	1. When I do things, my mind wanders off and I’m easily distracted. 2. When I’m doing something, I’m only focused on what I’m doing, nothing else.
			*Observing*	1. I notice changes in my body, such as whether my breathing slows down or speeds up. 2. I pay attention to whether my muscles are tense or relaxed.
			*Describing*	1. I’m good at finding words to describe my feelings. 2. I can easily put my beliefs, opinions, and expectations into words.
			*Non-judgmental Acceptance*	1. I criticize myself for having irrational or inappropriate emotions. 2. I tell myself that I shouldn’t be feeling the way I’m feeling.
The Mindfulness-Based Relapse Prevention Adherence and Competence Scale (e.g., Chawla et al., 2010)	Individuals who completed inpatient or intensive outpatient substance abuse programs (*N* = 93)		*Adherence (adherence to individual components of MBRP and discussion of key concepts)*	Adherence: Discussion of key concepts 1. Noticing/awareness of current experience. To what extent do therapists encourage noticing and being aware of present-moment experience? 2. Acceptance of current experience. To what extent do therapists encourage bringing curiosity and a non-judgmental attitude to whatever arises in the present moment, regardless of whether it is pleasant, unpleasant, or neutral? 3. Acceptance versus Aversion. To what extent do therapists introduce the differences between relating to one’s experiences from a standpoint of acceptance as opposed to aversion? 4. Acceptance versus Action. To what extent do therapists discuss the importance of stepping out of auto-pilot (pausing, taking a breathing space, evaluating one’s choices etc.) as a means of engaging in mindful action (responding vs. reacting, making choices that are in one’s best interest), and/or to what extent do therapists describe the relationship between acceptance and skillful/mindful action?
		Therapist style/approach 1 = Low 5 = High Overall therapist performance 1 = Not satisfactory 5 = Excellent	*Competence (ratings of therapist style/approach and performance)*	Competence: Therapist style/approach 1. *Inquiry*: Therapists’ ability to elicit and respond to both verbal and non-verbal feedback. 2. *Attitude*: Therapists’ ability to model and embody the spirit of mindfulness. 3. *Use of key questions*: The overall extent to which the therapists used key questions in eliciting discussion about exercises and home practice. 4. *Clarifying expectations*: The extent to which the therapist addresses and clarifies ideas and misconceptions about mindfulness meditation. Competence: Overall therapist performance 1. How would you rate the overall quality of the therapy in this session? 2. How would you rate the ability of the therapists to work as a team? 3. How would you rate the ability of the therapists to keep the session focused and on topic? 4. Please rate the overall quality of delivery of the meditation exercises.
The Self-Comparison Scale (e.g., Neff, 2003)	Undergraduate Educational Psychology students (*N* = 391)	1 = Almost never 5 = Almost always	*Self-kindness*	1. I try to be understanding and patient toward those aspects of my personality I don’t like. 2. I’m kind to myself when I’m experiencing suffering.
	Undergraduate Educational Psychology students (*N* = 232)		*Self-judgment*	1. When I see aspects of myself that I don’t like, I get down on myself. 2. When times are really difficult, I tend to be tough on myself.
	Buddhist participants (*N* = 43)		*Common humanity*	1. When I feel inadequate in some way, I try to remind myself that feelings of inadequacy are shared by most people. 2. When I’m down and out, I remind myself that there are lots of other people in the world feeling like I am.
			*Isolation*	1. When I fail at something that’s important to me I tend to feel alone in my failure. 2. When I think about my inadequacies it tends to make me feel more separate and cut off from the rest of the world.
			*Mindfulness*	1. When something upsets me I try to keep my emotions in balance. 2. When I’m feeling down I try to approach my feelings with curiosity and openness.
			*Over-identification*	1. When something upsets me I get carried away with my feelings. 2. When I’m feeling down I tend to obsess and fixate on everything that’s wrong.
The Self-Other Four Immeasurables Scale (e.g., Kraus and Sears, 2009)	College students (*N* = 124)	1 = Very slightly or not at all 2 = A little 3 = Moderately 4 = Quite a bit 5 = Extremely	*Positive qualities toward self*	Friendly – toward myself Joyful – toward myself Accepting – toward myself Compassionate – toward myself
			*Positive qualities toward others*	Friendly – toward others Joyful – toward others Accepting – toward others Compassionate – toward others
			*Negative qualities toward self*	Hateful – toward myself Angry – toward myself Cruel – toward myself Mean – toward myself
			*Negative qualities toward others*	Hateful – toward others Angry – toward others Cruel – toward others Mean – toward others

It is indeed interesting to note the differing viewpoints and interpretations of mindfulness. Our own proposition, likewise, also adds credence, providing another comprehensive interpretation of the nature of mindfulness. Despite the complexity of viewpoints and interpretations and the quest for us to add theoretical contributions, we can surmise that mindfulness is *purposive* and *meaningful*, reflecting a person’s temperament, personality and, more importantly, his/her state of mind. This theoretical positioning of mindfulness, as concurred by Western scholars (e.g., Chiesa et al., 2011; Keng et al., 2011; Treanor, 2011; Bowlin and Baer, 2012; Hjeltnes et al., 2015), emphasizes two fundamental tenets:

i. A person’s experience of a *present state*, reflecting clear focus and personal contentment, may serve to instill an internal state of calmness, ease, and clarity.
ii. Experience of mindfulness, in its truest sense, may yield a number of *meaningful outcomes*, such as improvement in positive emotions (e.g., happiness) and personal functioning (e.g., performance in a subject matter), and weakening in negative emotions (e.g., anxiety).

In essence, the study of mindfulness has involved scholars from the United States, the United Kingdom, Europe, Australia, etc. (e.g., Baer et al., 2008; Sauer et al., 2011; Kabat-Zinn, 2015). Despite this collective interest, we purport that inconsistency is still evident in terms of a common definition and understanding of mindfulness. Why is this the case? Despite the effectiveness and robustness of factorial techniques (Schumacker and Lomax, 2004; Kline, 2011), it can be said that quantitative representations (e.g., a four-factor model) are somewhat limited and do not, in this case, provide comprehensive evidence of the nature of mindfulness. A factorial-derived mapping of mindfulness, from our point of view, is somewhat limited and too simplistic for interpretation and in-depth account of its structure. The crux of our argument then is that mindfulness encompasses much more than just a simple definition (e.g., say… a person’s psychological state of ease), which could simply espouse “a persons’ psychological state of ease,” or “a person’s experience of reflection and self-awareness.”

Interestingly, in the Western literature (e.g., a Google search), another terminology also coincides with the concept of mindfulness – namely, in this case, the concept of *meditation*, also known as *meditation practice*. So, from this introduction, what is meditation or meditation practice? From a general point of view, there are many different types of meditation practice – for example: breath-awareness meditation, visualization meditation, and mantra-based meditation^1^. From a more technical point of view, reflecting the importance of Buddhist teaching, Loden (1996) defines meditation as “thoroughly and deeply acquainting the mind with objects of virtue. Because virtuous minds are by nature happy and the source of future happiness, each time that you engage in meditation further happiness is brought into your life” (p. 23). In a similar vein, Olendzki (2009) considers meditation as being the “sustained consideration or thought upon a subject.… As such, it is always an exercise of ordered conceptual contemplation, involving the systematic and disciplined use of language, symbol, and concept” (p. 37). Kabat-Zinn (2015) likewise defines meditation as being “the systematic and intentional cultivation of mindful presence, and through it, of wisdom, compassion, and other qualities of mind and heart conducive to breaking free from the fetters of our own persistent blindness and delusions” (p. 1482). From this brief account, we could say that meditation practice (e.g., “seated meditation practice”) is intentional, enabling a person to seek positive experience of calmness, concentration, and emotional balance. In our own teaching of meditation practice at university, for example, we teach and engage students in a practice known as “walking meditation practice.” Students would, in this case, recite the Buddhist sutras as they “walk” in a straight line or in a circle, paying close attention to their breathing.

We advocate and contend, indeed, that both mindfulness and meditation practice are two interrelated, but distinct concepts. As practitioners and researchers of mindfulness from an Eastern perspective, we argue for the following interpretation: that meditation practice (e.g., walking meditation) acts a practical mechanism, which would then facilitate and enable the personal experience of mindfulness (e.g., reaching a state of self-actualization). In other words, differing from scholarly previous conceptualizations and interpretations, we align our deliberation with those established in Buddhist texts (e.g., “*Meditations on the path to enlightenment*”) (Loden, 1996). From this consideration, we argue that meditation is an intentional applied personal practice whereas, in contrast, mindfulness is the acquired knowledge and experience that a person subsequently attains. From this stipulation, we argue that it is somewhat erroneous to make statements such as, “I’m practicing mindfulness right now…” and “I am experiencing meditation at the moment….” Supporting our theorization is another interesting terminology, coined as *mindfulness meditation* (Kristeller, 2007; Tang et al., 2007; Bauer-Wu, 2010). What is mindfulness meditation? According to Kristeller (2007), mindfulness meditation, also referred to as “Vipassana practice” and “insight meditation,” is primarily concerned with the cultivation of a person’s “ability to bring a non-judgmental sustained awareness to the object of attention rather than cultivating focused awareness of a single object, such as a word or mantra, as occurs in concentrative meditation. Mindfulness meditation may utilize any object of attention – whether an emotion, the breath, a physical feeling, an image, or an external object-such that there is more flexibility in the object of awareness than there is in concentrative meditation and such that the object may shift from moment to moment” (p. 393). We appreciate and concur with this viewpoint of mindfulness meditation as indeed, upon reflection, we teach and practise this personal approach. For us, in our teaching, mindfulness meditation may entail a focus on and the visualization of Buddha during our meditative practice.

Mindfulness meditation then, from this consideration, is a style of meditation (Tang et al., 2007; Bauer-Wu, 2010), similar to that of concentration meditation, mantra meditation, and guided meditation. As a “mind control,” or a “training” technique, according to Kristeller (2007), mindfulness meditation may assist in the facilitation of the achievement of “physical relaxation, emotional balance, behavioral regulation, and changes in self-judgment, self-awareness, and relationship to others” (p. 395). As we discuss later in the article, mindfulness meditation is quite appropriate and potent, coinciding with the teaching of Buddhism and Confucianism – in other words, as we attest, mindfulness meditation is an applied practice, which may result in the achievement in understanding and experience of mindfulness.

## Contemporary Understanding and Research Development: Introduction of Proposition

At present, from our synthesis and review of the literature (e.g., Baer et al., 2008; Sauer et al., 2011; Kabat-Zinn, 2015), mindfulness is analogous to that of a person’s *meditational state*. From a practitioner’s point of view, mindfulness is concerned with an internal state of “calmness,” “ease,” and/or “relaxation.” By the same token, mindfulness is *not* concerned with a person’s ability or inability to be “mindful” of a situation and/or an event – for example, statements such as “I am mindful that we are late for our next appointment” and “I need to be mindful that his grandfather recently passed away” may, in this sense, reflect a person’s *cognizance* and/or *attentiveness* of a contextual situation. This consciousness does not, in our view, equate to meaningful understanding and/or experience of mindfulness. Mindfulness is more than just attentiveness and may delve into other complex facets. In this analysis, as we have argued, it is somewhat limited to perceive and interpret mindfulness as simply a psychological state of attentiveness, reflection, self-awareness, etc.

**FIGURE 2:**
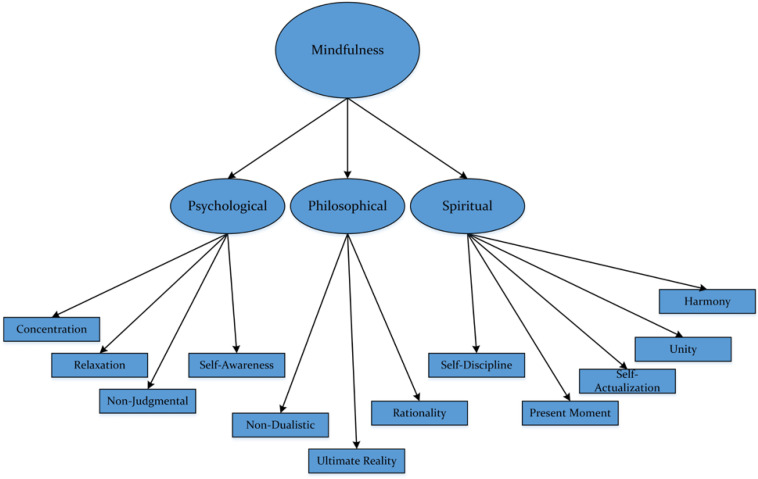
Conceptualization of holistic model of mindfulness.

Research development in the area of mindfulness is evolving and ongoing. One notable line of inquiry is, of course, concerned with theoretical, methodological, and empirical contributions to the elucidation and understanding of the nature of mindfulness. Ultimately, what is mindfulness and how does one perceive this on a daily basis? Our collaborative research development over the past 5 years, cross-institutionally and cross-culturally, in positive psychology (e.g., optimization: Phan et al., 2019c, 2020c) has led to our keen collective interest to consider a proposition of a holistic model of mindfulness. At the time of preparation and write-up of our book, titled “*Teaching*, *Learning and Psychology*,” we briefly introduced this holistic model and contended that it offers a more inclusive definition of mindfulness. Our proposed model, as shown in Figure 2, is innovative and differs from existing representations that largely emphasize the importance of Western ideas and theoretical stance (e.g., Baer et al., 2008; Sauer et al., 2011; Kabat-Zinn, 2015).

### The Importance for More Inclusiveness

Literatures delving into Confucianist teaching (Yao, 2000; Havens, 2013) and Buddhist philosophy (Yeshe and Rinpoche, 1976; Master Sheng Yen, 2010) have so much to offer, especially in terms of providing theoretical understanding into the nature of mindfulness. Many scholars, from Eastern contexts, would argue that existing research development into the nature of mindfulness from Western settings is somewhat limited. Psychological emphasis (e.g., state of relaxation), alone, is restricted and does not take into account the gamut of factors and/or facets that could illuminate the true “essence” of mindfulness. Having said this, however, we do note that in recent years, some Western scholars (e.g., Carmody, 1984; Carmody et al., 2008; Mutter, 2014; Lazaridou and Pentaris, 2016) have incorporated and have placed emphasis on non-psychological entities – for example, the importance of spirituality within the realm of mindfulness. Our own research development and professional experiences (e.g., teaching mindfulness at university), for example, have led us to strongly advocate for the inclusion of attributes that espouse to the teaching of Confucianism (Yao, 2000; Havens, 2013) and Buddhism (Yeshe and Rinpoche, 1976; Master Sheng Yen, 2010). In this analysis, meaningful understanding of mindfulness does not simply entail, for example, the experience of and appreciation for a relaxed state of mind. It is much more than this viewpoint, we believe. By the same token, not appearing to sound restrictive (e.g., restricting to Buddhism alone), we speculate that other specific epistemologies, philosophical beliefs, and rationales (e.g., faith in Hinduism) may also help to elucidate and add clarity to the study of mindfulness. In this analysis, we purport for a more holistic outlook by which different cultural interpretations could be offered to describe and explain the “totality” of mindfulness.

As shown in Figure 2, our proposition indicates intricacy and that, more importantly, there is the “merging” of both Western and Eastern ideas. This consideration, indeed, rejects the view that mindfulness is simply a psychological entity. Similarly, in line with this reasoning, we recently devoted a chapter in our forthcoming book, titled “*In search of sociocultural and psychological explanations of human agency: Western and Eastern insights for future development*,” which seeks to explore the complex issues of methodology. In other words, as we explore later in the article, the methodological issue of measurement and assessment is imperative, especially in terms of empirical validation of the proposed model of mindfulness.

### Proposed Model of Mindfulness

A model of mindfulness that is more inclusive, as shown in Figure 2, may espouse both Western (e.g., Baer et al., 2004; Davis et al., 2009) and Eastern (Nyanaponika Thera, 1972) ideas and theoretically-derived tenets. In this analysis, via means of focus-group discussions with Taiwanese scholars who specialize in knowledge, experience, and teaching of Asian cultural studies (e.g., Chinese History), mindfulness, and meditation, we theorize and postulate three components: *psychological component*, *philosophical component*, and *spiritual component*. This conceptualization, in particular, purports that mindfulness is much more than just testament of a person’s observational state of cognition and/or behavior. Our main premise is that, unlike research studies in Western contexts, there are many “non-observable” attributes, which may define mindfulness. In this analysis, we contend that Likert-scale measures and/or open-ended surveys, alone, do not capture the essence of mindfulness.

So, what is our proposition? Table 2, referenced from Phan and Ngu’s (2019) recent publication, provides a summation of description of the three components and their respective attributes:

**TABLE 2:** A Summary of psychological component, philosophical component, and spiritual component.

**Mindfulness**		
**Themes**	**Indicators**	**Definition**
**Psychological**		
	Concentration	This indicator emphasizes a person’s mindset, which is resolute and focused. Mindfulness, in this sense, recognizes the importance of *focus*, which is direct, purposive, and directs toward a particular know. For example, concentration may involve a person’s focus on a particular keyword (e.g., happiness), or a thing in the contextual environment (e.g., a flower) with ease. Concentration, in this case, does not entail deviation of a person’s mindset and/or focus – that is, concentration does not permit a person’s mind to “wonder off.”
	Relaxation	This indicator recognizes the importance of a person’s *psychological* and *physiological attributes*, which are at ease. Mindfulness, in this case, enables and facilitates a person to “ease” his/her emotions, feelings, and physiological functioning. Relaxation, in this case, would free the mind from any provoking thoughts, emotions, and/or actions. A mindset that is “relaxed” would help a person to reach a state of enlightenment, which then may result in harmony, peace, and love. Non-relaxation, of course, indicates a confused mindset that, correspondingly, yields negative heightened psychological and physiological attributes.
	Non-judgmental	This indicator focuses on a person’s judgment of others and what this judgment entails. Judgments can be both positive (e.g., Sheng seems to get on with his friends) and negative (e.g., Ya-Chu does not seem to have any friends). Mindfulness, in this case, enables and ensures that a person is non-judgmental in his/her daily functioning – that is, he/she does not make judgments about others, regardless of their differences, backgrounds, situations, and personal circumstances. A non-judgmental mindset reflects a state of contentment, and the achievement in experience of unity and harmony.
	Self-awareness	This indicator emphasizes the importance of a person’s recognition and awareness of his/her own mindset, which in this case entails emotions, thoughts, actions, ease, and the contextual surrounding. Does one know, for example, that one is unhappy and experiencing a state of anxiety? And why is this the case? Mindfulness, in this case, enables a person to develop and to experience an internal state of cognizance, which may assist him/her to reach *enlightenment*. Lack of self-awareness, in contrast, reflects the inability to engage in and/or to experience mindfulness.
**Philosophical**		
	Non-dualistic	This indicator focuses on the importance of non-separation, and the fundamental point of essential oneness (i.e., wholeness, completeness, or unity). This emphasis on non-duality suggests that as individuals, we are all one at the deepest level of our existence. There is no such thing, in this analysis, of separation and/or diversity (e.g., this *versus* that, you *versus* me), – there is only one universal essence, and one reality by which we are all included. The physical body and mind become one – that is, mind and matters combine. The importance of oneness. When others experience, you feel pain as well. People + People Mind + Physical Mind + Mind
	Ultimate reality	This indicator contend that ultimate reality is the absolute nature of all things. As individuals, we use our observations, consciousness, and experience to define ordinary reality, which we hold as being truthful. Mindfulness, in part, focuses on the transcendence between the physical and the non-physical dimensions of our world. At the same time, engagement in mindfulness enables a person to recognize and to acknowledge the existence of an all-inclusive reality, by which all things are derived.
	Rationality	This indicator emphasizes the importance of reality, absolute in nature, which then determines a person’s course of action. As individuals, we act by reasons, which are in accord with the facts of reality. Irrationality, in contrast, reflects the undermining of one’s own mind and conviction to act in a rational manner. In choosing irrationality, a person then conveys the message that he/she lacks a rational mind. Mindfulness, in this sense, acknowledges our thought processes as sources that guide our convictions, values, goals, desires, and actions.
**Spiritual**		
	Self-discipline	This indicator focuses on a person’s ability to accomplish things, to regulate and train personal conduct, and to regulate personal feelings, emotions, and desires. This emphasis of self-discipline, of course, recognizes the importance of mental strength, which may involve personal resolve and persistence, to self-regulate and control one’s own behaviors, feelings, emotions, and desires. Mindfulness, in this sense, enables a person to develop and to experience an inner self, which then translates and/or reflects a state of discipline.
	Present moment	This indicator, interestingly, focuses on time, differentiating the past, the present, and the future. Buddhism, in this sense, does not recognize the past, nor does it recognize the future. Rather, the present moment is the only thing where there is no time. The present moment is always there, and it also serves as a meeting point between past and future. We can never access any other timeframe, other than the present time point. In this analysis, everything that ever happened and will ever happen can only happen in the present moment, and not at any other time points. The past and the future do not have distinctive realities – only the present moment can. Our memories of the past, and/or our consideration of the future are simply mental concepts. Meditation, in this sense, attempts to enable a person to become present oneself. When a person is in a state of “present moment,” he/she does not think of the past, nor does he/she consider of his/her future.
	Self-actualization	This indicator, similar to that of the concept of nirvana, emphasizes a person’s potential to become what he/she is capable of. In this sense, considering the perspective of Buddhism, self-actualization reflects a person’s desire and inner strength to reach “enlightenment.” Experiencing the “present moment,” in particular, assists a person to achieve a Buddha-nature state of perfection or tranquility, which is known as *satori*. Enlightenment, ultimately, indicates and/or reflects a person’s mind and personality merging with nature and reality. When this occurs, consequently as a result of engagement in mindfulness, one is able to experience a state of unity and harmony.
	Unity	This indicator places emphasis on the importance of bonding between individuals. The ideal positioning, through mindfulness, reflects an internal state of “many in body, one in mind” (i.e., we are all different, but share the same spirit). With a state of ease, mindfulness facilitates our thinking to consider unity of diversity, and to recognize that we all have to work collectively toward self-reformation, and to look out for each other for a better future. Ultimately, as humans, we are all followers of Buddha with the main desire to obtain *nirvana* (i.e., *enlightenmen*t).
	Harmony	This indicator emphasizes the importance of pleasant and non-contentious functioning between individuals, and/or between groups of people. Mindfulness, in this sense, reflects a desire to achieve *enlightenment* or *nirvana*, which then enables a person to experience harmony and peace. A state of harmony, via means of mindfulness, intricately associates with other qualities, such as *love*, *generosity*, *appreciation of others*, *sensitivity*, *forgiveness*, *kindness*, *respect*, *sympathy*, and *tolerance*.

• *Psychological Component*: concentration, relaxation, non-judgment, and self-awareness.
• *Philosophical Component*: non-dualistic, ultimate reality, and rationality.
• *Spiritual Component*: self-discipline, present moment, self-actualization, unity, and harmony.

In this analysis, we argue that mindfulness is rooted in a tripartite system of psychological, philosophical, and spiritual measures. In other words, we contend that this proposed tripartite system of mindfulness has both “scientific” (e.g., psychological measure?) and “non-scientific” (e.g., philosophical and spiritual measures?) measures, and may require engagement and usage of alternative non-traditional methodological approaches. In brief, as detailed in Table 2, we have three components:

#### Philosophical Component

The philosophical component of mindfulness focuses on the *epistemology and exploration* of the contextual nature of mindfulness. Epistemology, in this case, is concerned with a persons’ quest to seek understanding into the true “meaning” of mindfulness. What does mindfulness actually mean and, more importantly, what does it constitute? This component of mindfulness, from our rationalization, delves into the reading of Buddhist scriptures and places emphasis on a person’s philosophical stance. In other words, we contend that individual experience of mindfulness, via means of meditation would direct and facilitate a person to engage in philosophical “pondering.” This “philosophical pondering” considers a person’s reflection and willingness to explore comparative and contrasting scenarios, viewpoints, and propositions.

A philosophical viewpoint of mindfulness, from our summation (Table 2), emphasizes the importance of self-reflection, introspection, and contemplation. Experience of mindfulness, in this sense, delves into a person’s mindset and his/her relationship with nature and reality. For example, in his/her state of philosophical pondering, a person may contemplate about the universe and where he/she is at. Ultimately, the philosophical component of mindfulness espouses a perceived sense of openness, guiding a person toward appreciation for life and of life itself. In essence, the philosophical component of mindfulness has the potential to instill philosophical reasoning, enabling a person to question his/her own existence, contextual surroundings, and/or personal life experiences.

Aside from the mentioned testament, it is also a plausibility for a person to seek philosophical understanding about the nature of mindfulness. Philosophically, for example, what is mindfulness? What does it mean, theoretically, when a person is in a philosophical state of reasoning? Do we, as a specific being, differ from other beings and, more importantly, can we coexist? These few questions are examples, which may focus on the nature of the philosophical component of mindfulness. As physical beings, experiences of mindfulness enable us to question our own existence with nature and, by the same token, engaging in philosophical reasoning would permit us to acknowledge and recognize our own mortalities and rationalities.

#### Spiritual Component

The spiritual component of mindfulness focuses on the *importance of spirituality*. Spirituality, from an Eastern perspective, may encompass the true meaning of the afterlife and, of course, other life-related aspects that cannot be accounted for by the laws of physical sciences. The physical world, for example, defines a linear time point: past, present, and future (Phan et al., 2020b). We think about the past, live in the present moment, and consider our future outlooks. From this understanding, situated within a larger system of change, a person may seek to understand about his/her holistic being. What does this actually entail?

Experience of mindfulness, from our point of view, may enable a person to seek understanding into his/her presence in this universe. The physical body exists within the realm of a person’s lifetime – that is, from birth to death. However, spiritually, we also place emphasis on the “human spirit” or a “person’s soul.” From this understanding, we contend that experience of mindfulness would offer opportunities for individuals to reflect and to ponder about other “realms” of reality. Spiritually, a person could ponder and seek to understand the meaning of the afterlife – for example, what happens when a person moves on from this living world? Where does his/her soul reside? Where happens to his/her state of consciousness? These questions are examples that may reflect the complex nature of spirituality of mindfulness.

Self-awareness, indeed, is a state by which a person would seek to understand and appreciate. Personal self-awareness, in this case, would enable a person to situate his/her mindset to the present moment with the focus being on a quest to strive for enlightenment. Time, as we previously described, is an important entity by which only the present time point counts. In this sense, appreciation of spirituality enables a person to contemplate the meaning of peace, harmony, connectedness, and unity. By the same token, of course, experience of mindfulness would purposively allow a person to delve into the meaning of *transcendence*. Transcendence, in this case, is related to a person’s cognizance that there is a division between the living world and the non-living world.

#### Psychological Component

The psychological component of mindfulness focuses on the *importance of a person’s psychological mindset.* This emphasis, of course, closely relates to a person’s state of consciousness, delving into a few notable attributes – for example: (i) a person’s state of concentration, (ii) a person’s ability to remain non-judgmental, (iii) a need to be cognizant of the contextual surrounding and of himself/herself, and (iv) to recognize the importance of ease, calmness, and serenity.

In essence, the psychological component of mindfulness places emphasis on a person’s psychological mindset to be able to self-regulate his/her thoughts and behaviors. Experience of mindfulness, in this case, would enable a person to possess and exhibit an appropriate temperament, such as the ability to remain calm and to be non-judgmental of nature itself. In other words, from the perspective of Buddhism, mindfulness does not place emphasis on encouragement for individuals to make judgments and/or to be judgmental of others – for example, “that is a pretty dress that you are wearing.” Everything in nature is as it is and, importantly, there is no valence – that is, the positives versus the negatives.

Experience of mindfulness, psychologically, entails an unwaivered mindset by which a person is able to remain on task in terms of his/her attention. Recently, in a study involving Taiwanese university students, we introduced and explored a psychological concept, which we termed as “personal resolve.” Personal resolve differs from a state of resilience (Martin and Marsh, 2006) and/or self-determination (Deci and Ryan, 2008), and “considers interestingly the importance of a person’s mental resolute and “unwavering focus” to stay on task without any uncertainty or reservation to achieve optimal best. In this analysis, … personal resolve focuses on a person’s conviction that his/her choice, positioning, and action are indeed correct, despite what others may say” (Phan et al., 2020c). From this understanding, we contend that experience of personal resolve may indeed reflect the psychological nature of mindfulness, especially in terms of a person’s state of concentration.

#### In Totality: A Multifaceted Structure of Mindfulness

In total, what can we take away from the preceding sections? Our main premise, in this case, is that mindfulness is complex and may espouse a multifaceted structure, consisting of three major components: psychological, spiritual, and philosophical. This rationalization, in part, rejects existing research inquiries and theorizations from Western contexts, which place strong emphasis on different psychological themes. Stemming from personal experiences and the teaching of Buddhism and Confucianism, we argue that mindfulness is more than just testament and reflection of psychological processes (e.g., a person’s temperament). Rather, as a point of totality, we purport that perceived understanding of mindfulness encompasses the gamut of human experiences, consciously and subconsciously.

The distinction of our proposed model of mindfulness lies in its merging of Eastern and Western ideas, reflecting inclusiveness of philosophical, spiritual, and psychological attributes. At the same time, of course, we contend that our proposition places emphasis on the notion of “universality,” which encompasses both scientific (e.g., state of concentration) and non-scientific (e.g., state of transcendence) inquiries. This consideration emphasizes the use of personal reasoning and philosophical psychology to seek understanding into the intricate nature of mindfulness. By all account, the proposition detailed in Figure 2, as Phan and Ngu (2019) recently introduced, is conceptual and would require rationalization and continuing research development. Speculative, however, it is plausible for us to draw in differences between individuals. For example, novice practitioners of meditation may simply experience an internal state of relaxation and calmness and, eventually, come to recognize the importance of his/her focus of concentration (e.g., on a subject). Novice learners of mindfulness, from our point of view, would not necessarily have the skills and/or experiences to reflect on their inner selves, and/or to view everyone with a sense of fairness. In contrast from this, more experienced practitioners of meditation (e.g., Buddhist monks/nuns) would have advanced understanding and appreciation of mindfulness, differentiating themselves by their achievements of *enlightenment* and *satori*. Moreover, advanced individuals of mindfulness would recognize the intricate “bond” between different beings and nature. An internal state of *nirvana*, in this case, would allow individuals to develop and flourish in different types of personal attributes, such as love, generosity, forgiveness, kindness, and respect for all different beings. In other words, in-depth and personal experience of mindfulness from our point of view may instill a higher-order “philosophical mindset,” resulting in a person’s appreciation and acknowledgment that there is no distinction and/or differentiation between different beings in nature (e.g., human beings, birds, dogs, etc.). In essence, from this testament, the significance of our proposed multifaceted model of mindfulness lies in understanding that a person’s experience of mindfulness may entail different types of philosophical, spiritual, and religious sentiments for contemplation – for example: what happens when a person passes on from this living world? what makes a person different from another person but, more importantly, should this “difference” affect his/her attitude, respect, viewpoint, etc. for that person?

## Testament of Mindfulness as Part of Positive Psychology

By all means, development into the holistic representation of mindfulness, taking into account philosophical component, psychological component, and spiritual component is evolving (Figure 2). What we have presented so far in this article is introductory, and entails the following:

• Advocation for a complex, multifaceted structure of mindfulness, which reflects a combination of both Western and Eastern ideas. This emphasis is significant, advocating for the sharing of knowledge and the acceptance of cross-cultural comparison in viewpoints and epistemologies.
• Consideration of consciousness and the subconsciousness and, by the same token, acknowledgment of both scientific and non-scientific attributes, which could account for the complex nature of mindfulness.
• Acknowledgment that practice of meditation gives rise to a state of mindfulness, differentiating novice from experienced practitioners. Meaningful understanding of mindfulness, in this case, may consist of testament of evidence of philosophical experience, psychological experience, and/or spiritual experience.

**FIGURE 3:**
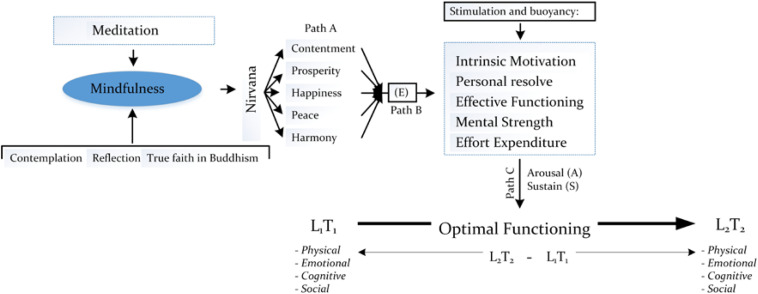
Conceptualization of mindfulness and optimization.

It is sound and logical to consider mindfulness as an entity of positive psychology (Csíkszentmihályi, 1990; Seligman, 1999; Seligman and Csíkszentmihályi, 2000). How do we rationalize this theoretical positioning – *that mindfulness could be part of the repertoire of positive psychology?* To answer this question, let us refer back to the concept of optimal best (Martin, 2011; Liem et al., 2012; Phan et al., 2016) and, in particular, the active process of optimization (Fraillon, 2004; Phan et al., 2019c, 2020b). In a recent book chapter, Phan et al. (2019d) discussed this association in detail – that mindfulness could act as an optimizing agent, which would in turn facilitate the accomplishment of optimal best. We refer to Phan et al.’s (2019d) rationalization for discussion in this section. To assist with this postulation (i.e., the relationship between mindfulness and optimization), we have developed a conceptual model, as shown in Figure 3.

The proposition detailed in Figure 3 is innovative, illustrating the potentiality for perceived experience of mindfulness to act as an antecedent in the process of optimization. As an example, with reference to Figure 3, a person engages in Buddhist meditation, which would result in his/her understanding and experience of mindfulness – in this case, this understanding and experience of mindfulness is indicated by the person’s reflection, contemplation, and testament of true faith in the philosophy of Buddhism. We propose that this development in understanding and experience of mindfulness, in turn, would act to instill a suite of *Buddhist-related attributes* for acknowledgment and recognition – for example: personal contentment or satisfaction, the prosperity of health functioning, and a perceived sense of happiness, peace, and harmony. These Buddhist-related attributes, as positive and proactive concepts, from our conceptualization, may act as sources of information to initiate an appropriate level of energy, E, for further enactment (Phan et al., 2019c, 2020b). In accordance with recent development of the process of optimization, we postulate that a high level of energy would result in the activation and buoyancy of different psychological attributes (e.g., effort expenditure), resulting in arousal and the sustaining of a state of functioning (e.g., happiness).

**FIGURE 4:**
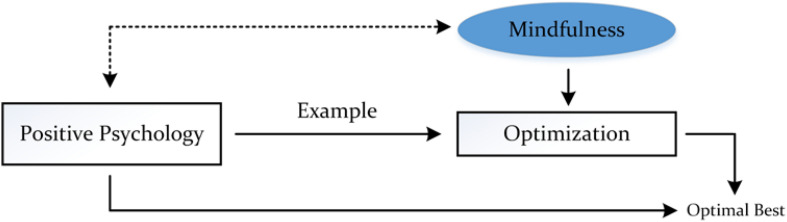
Conceptualization of mindfulness and positive psychology.

Note: We have drawn a dotted line to depict the direct association between positive psychology and mindfulness. However, despite this recording, we propose that the relationship between the two concepts is evident via the process of optimization (i.e., non-dotted lines).

The above example is insightful as it helps to elucidate the potential relationship between our proposed model of mindfulness and the paradigm of positive psychology (Csíkszentmihályi, 1990; Seligman, 1999; Seligman and Csíkszentmihályi, 2000), which in this case is related to the process of optimization (Fraillon, 2004; Phan et al., 2019c, 2020b) and, subsequently, the achievement of optimal best. This depiction is summarized in Figure 4 where we have the following: (i) indicative of the paradigm of positive psychology is the achievement of optimal best (e.g., positive emotions), L_2_, and the process of optimization, and (ii) resulting from the process of optimization, which mindfulness may act as an optimizing agent, is the achievement of optimal best, L_2_, Our proposition, as shown, posits the indirect influence of mindfulness on optimal best, via the process of optimization. In other words, rather than a direct association (i.e., mindfulness^®^ optimal best), we argue that mindfulness is prevalent via means of its optimizing role.

## Implications for Consideration: Theoretical and Methodological Contributions

Scientific inquiries, such as research undertakings that focus on students’ academic performance outcomes are direct and may involve the use of conventional methodological approaches (e.g., a two-group experimental design). Complexities arise, however, when we have inquiries that do not conform to and/or situate within the physical world. In this analysis, measuring and assessing a psychological, factorial structure of mindfulness is relatively straightforward, which may involve Likert-scale responses that are analyzed within the framework of CFA techniques (e.g., Baer et al., 2004, 2008; Cardaciotto et al., 2008). Having said this, though, validating our proposed multifaceted model of mindfulness is more difficult, to the point where it may be perceived as being improbable. Traditional methodological means, in this analysis, may not be adequate. For example, how would we validate the spiritual component of mindfulness? Understanding the true meaning of nirvana (Phan and Ngu, 2019; Phan et al., 2019d), in this analysis, is somewhat difficult to achieve, given the fact that it would be infeasible to quantify and measure.

In our recent research development, we devoted a chapter in which we explored in detail the issue of what is termed as “methodological appropriateness” (Phan et al., 2019a). Phan et al. (2019c), in their conceptual article, introduced this term, which is defined as the development of an *appropriate* methodological design that would enable the accurate measurement and assessment of a process or an outcome (e.g., optimization). The authors’ rationale, in this case, is that inappropriate methodological designs would produce inaccurate results and/or misconstrued interpretations. On this basis, our chapter also explored the importance of appropriate methodological designs for usage to measure and assess non-scientific inquiries. In the present context, for example, emphasis of methodological appropriateness could involve the use of *focus-group discussions and sharing of experience*, *in situ observations*, and *reflective writing* to seek relevant insights into the multifaceted nature of mindfulness.

We perceive that certain aspects of our proposed model of mindfulness are “esoteric” and “mythological,” which would make it somewhat difficult to ascertain some form of scientific evidence. For example, experience of satori in which a person is able to reach a Buddha-nature state of tranquility would, in this sense, be difficult to validate. A perceived sense of satori, according to experienced practitioners of meditation, is “internalized” and not yield observational information for documentation. In a similar vein, testament of nirvana, that is a state of enlightenment, is somewhat difficult to document and to ensure accuracy for the purpose of comparison and consistency. Our mentioning of “non-scientific” attributes (e.g., the attribute of “ultimate reality”: Figure 2), in this case, emphasizes the importance of non-physical, non-contextual, and non-cognizant experiences.

We contend that at any moment in time, a person’s state of consciousness and engagement with the physical world serve to explain his/her experience of mindfulness. Personal experience, arising from maturity and ongoing practice of meditation, may provide grounding for individuals to develop “insights” into their sub-consciousness, enabling them to acquire esoteric and mythological experiences. Rather than focusing on conventional methodologies (e.g., a two-group experimental design), we propose the use of Eastern, non-traditional epistemologies, which could offer evidence and in-depth understanding of different personal esoteric experiences. Some Taiwanese colleagues that we know of, for example, engage in non-traditional epistemologies such as: (i) reflecting on their acquired wisdom about the world and life, in general, (ii) documenting their deep, meaningful insights into the living world, and (iii) to consider thoughts, behaviors, and actions that are “higher-order.” These methodological positionings, of course, are relatively unfounded and may lack credibility, scientifically. However, despite this contentious methodological approach, some Taiwanese scholars and experienced practitioners of meditation have attested to the fact that their esoteric, non-conventional experiences of mindfulness have helped them in their daily lives. In a similar vein, we recommend for the inclusion of contributions, theoretical, conceptual, empirical, and/or methodological, from other sociocultural settings. It would be of interest, in this analysis, for us to consider and incorporate other philosophical faiths – for example: how does true faith in Hinduism account for understanding and personal experience of mindfulness? how does an Indigenous group’s particular cultural esoteric practice assist in the development of understanding and experience of mindfulness?

## Conclusion

Positive psychology, as extensive writings have shown, is an interesting paradigm for reading. At the same time, of course, educators and researchers have used positive psychology to structure and design various programs for implementation, which would ultimately result in the determent of pathologies as well as the promotion of positive conditions and positive life experiences. Our own research development over the past 5 years, likewise, has made extensive theoretical, methodological, and empirical contributions to the study of positive psychology. One notable aspect of our research, which we share in this article is the theorization and development of a proposed multifaceted model of mindfulness that takes into consideration both Western and Eastern ideas.

We contend that mindfulness is a complex concept that scopes different themes and attributes, scientifically and non-scientifically. Our theoretical contention, as explored in this article, is that mindfulness (i.e., our proposed multifaceted model) could coincide with and support the study of positive psychology. In particular, adhering to the theory of optimization, we postulate that mindfulness could act as an “optimizing agent,” which then would assist in the facilitation of a person’s achievement of optimal best (Phan et al., 2017, 2019d; Phan et al., 2020b). This consideration is interesting, reflecting our use of philosophical psychology, personal reasoning, and extensive experiences in teaching and research development of optimization and mindfulness. Aside from the postulation that mindfulness is closely associated with positive psychology, we also offer methodological issues that are of significance for continuing research.

## Author Contributions

HP and BN were responsible for the conceptualization, literature search, and write-up of this manuscript. SC, LW, S-YS, R-YL, J-HS, and H-WW contributed equally in terms of conceptualization, discussion, literature search, and philosophical reasoning with specific reference to Buddhism and Buddhist mindfulness.

## Conflict of Interest

The authors declare that the research was conducted in the absence of any commercial or financial relationships that could be construed as a potential conflict of interest.

## Footnotes

1 https://chopra.com/articles/whats-the-difference-between-meditation-and-mindfulness
